# Localised genetic heterogeneity provides a novel mode of evolution in dsDNA phages

**DOI:** 10.1038/s41598-017-14285-0

**Published:** 2017-10-23

**Authors:** Damian J. Magill, Phillip A. Kucher, Victor N. Krylov, Elena A. Pleteneva, John P. Quinn, Leonid A. Kulakov

**Affiliations:** 1 Queen’s University Belfast, School of Biological Sciences, Medical Biology Centre, 97 Lisburn Road, Belfast, BT9 7BL Northern Ireland; 2 Department of Microbiology, Laboratory for Genetics of Bacteriophages, I.I. Mechnikov Research Institute for Vaccines and Sera, Moscow, Russia

## Abstract

The Red Queen hypothesis posits that antagonistic co-evolution between interacting species results in recurrent natural selection via constant cycles of adaptation and counter-adaptation. Interactions such as these are at their most profound in host-parasite systems, with bacteria and their viruses providing the most intense of battlefields. Studies of bacteriophage evolution thus provide unparalleled insight into the remarkable elasticity of living entities. Here, we report a novel phenomenon underpinning the evolutionary trajectory of a group of dsDNA bacteriophages known as the *phiKMVviruses*. Employing deep next generation sequencing (NGS) analysis of nucleotide polymorphisms we discovered that this group of viruses generates enhanced intraspecies heterogeneity in their genomes. Our results show the localisation of variants to genes implicated in adsorption processes, as well as variation of the frequency and distribution of SNPs within and between members of the *phiKMVviruses*. We link error-prone DNA polymerase activity to the generation of variants. Critically, we show trans-activity of this phenomenon (the ability of a *phiKMVvirus* to dramatically increase genetic variability of a co-infecting phage), highlighting the potential of phages exhibiting such capabilities to influence the evolutionary path of other viruses on a global scale.

## Introduction

Knowledge of evolutionary processes is one of the pillars of biology, and viruses have been paramount in our understanding of a number of such phenomena. Co-evolution, defined as a process of reciprocal adaptation and counter-adaptation between interacting species^1–3^, has been illuminated predominantly due to studies of phage-host systems. One such initial insight is that co-evolutionary scenarios can take place on the scale of up to 1.5 cycles of reciprocal change for phages such as T1, T2, T4, and T7^4–8^. These are due to the emergence of a bacterial genotype possessing resistance for which the phage is incapable of circumventing. Generally, such resistance manifests itself as alterations to cell surface constituents utilised by phages in the adsorption process of the infection cycle^9^. This situation has been described as asymmetrical, as one can argue that changes and even loss of a receptor can be achieved by multiple routes, whereas the mutational constraints imposed on phages being able to bind to modified or indeed a different receptor are higher^8^. As logical as this may sound, other co-evolutionary scenarios however, have shown the capability of phages to engage in extremely prolonged cycles of reciprocal change^10–12^. It seems clear that fundamental differences in the biology of phages are contributing to varying ecological patterns and understanding the underlying processes at play is of paramount importance.

The *phiKMVviruses* ^13^ are a group of ubiquitously distributed lytic dsDNA bacteriophages related to enterobacterial phage T7, some of which are utilised in a number of therapeutic preparations targeting the nosocomial pathogen *Pseudomonas aeruginosa* ^13,14^. The clinical and environmental contexts of these phages makes them a useful system of study for phage biology as a whole, but fundamental understanding of the behaviour of these viruses is additionally important due to their therapeutic potential.

In this paper, we report the discovery and initial characterisation of a novel phenomenon operating within members of the *phiKMVviruses*. This discovery reveals the evolutionary elasticity of these phages, highlighting their capability to potentially circumvent host resistant phenotypes on the co-evolutionary battlefield.

## Results and Discussion

**Figure 1:**
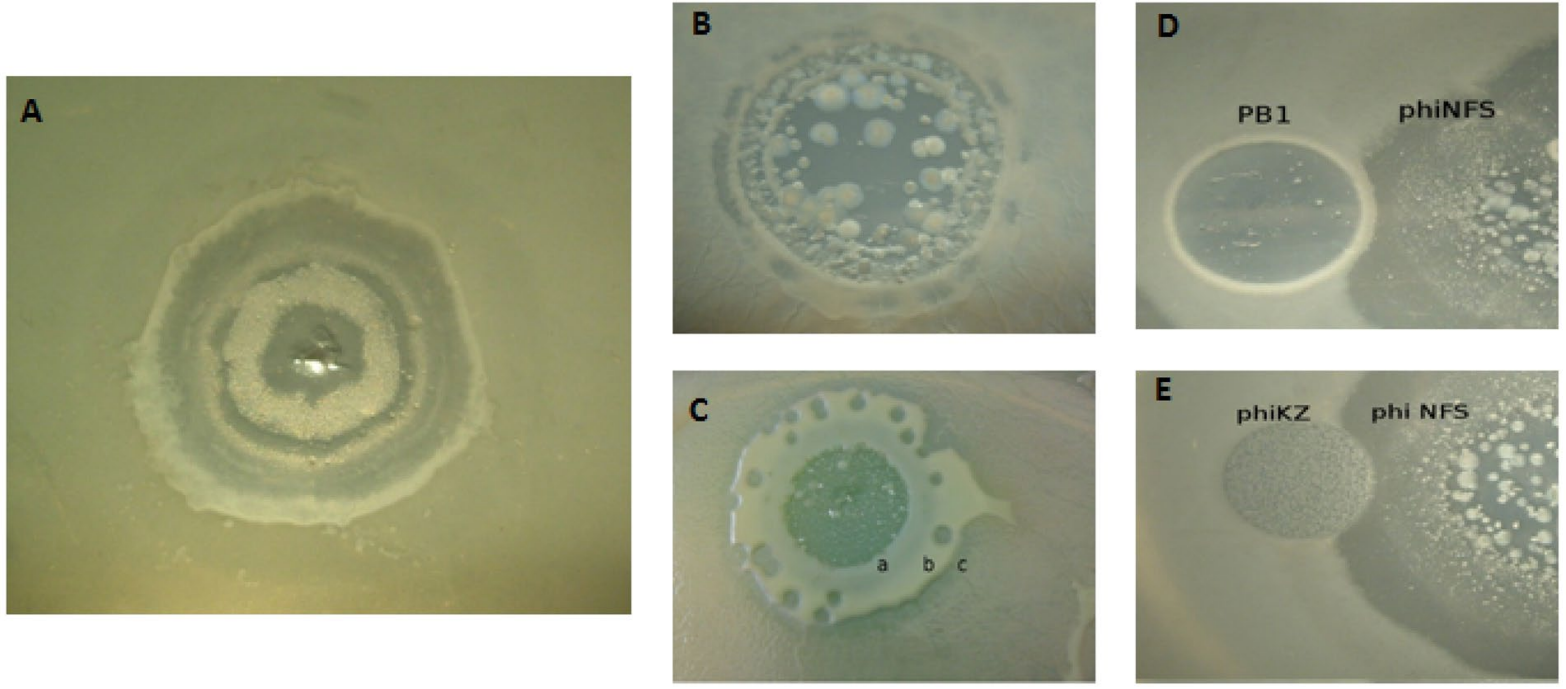
Unusual growth patterns/interactions of the *phiKMVvirus* phiNFS. Unique growth behaviours exhibited by phage phiNFS, including cyclic growth alternating between lysis and pseudolysogeny (**A**), growth of phiNFS on plaquing variants of *Pseudomonas aeruginosa* (**B**,**C**), and inhibition of phiNFS plaque boundaries by phages phiKZ and PB1 (**D**,**E**).

The study of the *phiKMVvirus* phiNFS, isolated from a Russian therapeutic phage preparation, revealed a broad host-range and unusual growth patterns. This included a cyclic interchange between phases of clear lysis along with regions of bacterial overgrowth indicative of pseudolysogeny (Fig. 1). On a bacterial lawn a typical plaque increases in size for as long as the host can support the replication and maturation of the phage at the plaque’s boundary. Growth finally ceases due to the lack of, or accumulation, of some factor necessary or inhibitory to the phage replication cycle. Virulent phages, such as those of the *Pbunavirinae* may produce circles of dense bacterial growth (pseudolysogenic state) at the plaque’s border. In phiNFS, we observed such a phenomenon, but with the difference that this phage showed a new adaptation to the bacterial lawn, permitting continued growth beyond the pseudolysogenic regions. In phiNFS, this is observed for a period of up to 7 days. The cyclic nature of growth between the dense pseudolysogens and lytic spots seems to reflect “adaptation” by phiNFS, which may be attributed to variants in adsorption associated genes observed in this work.

**Figure 2:**
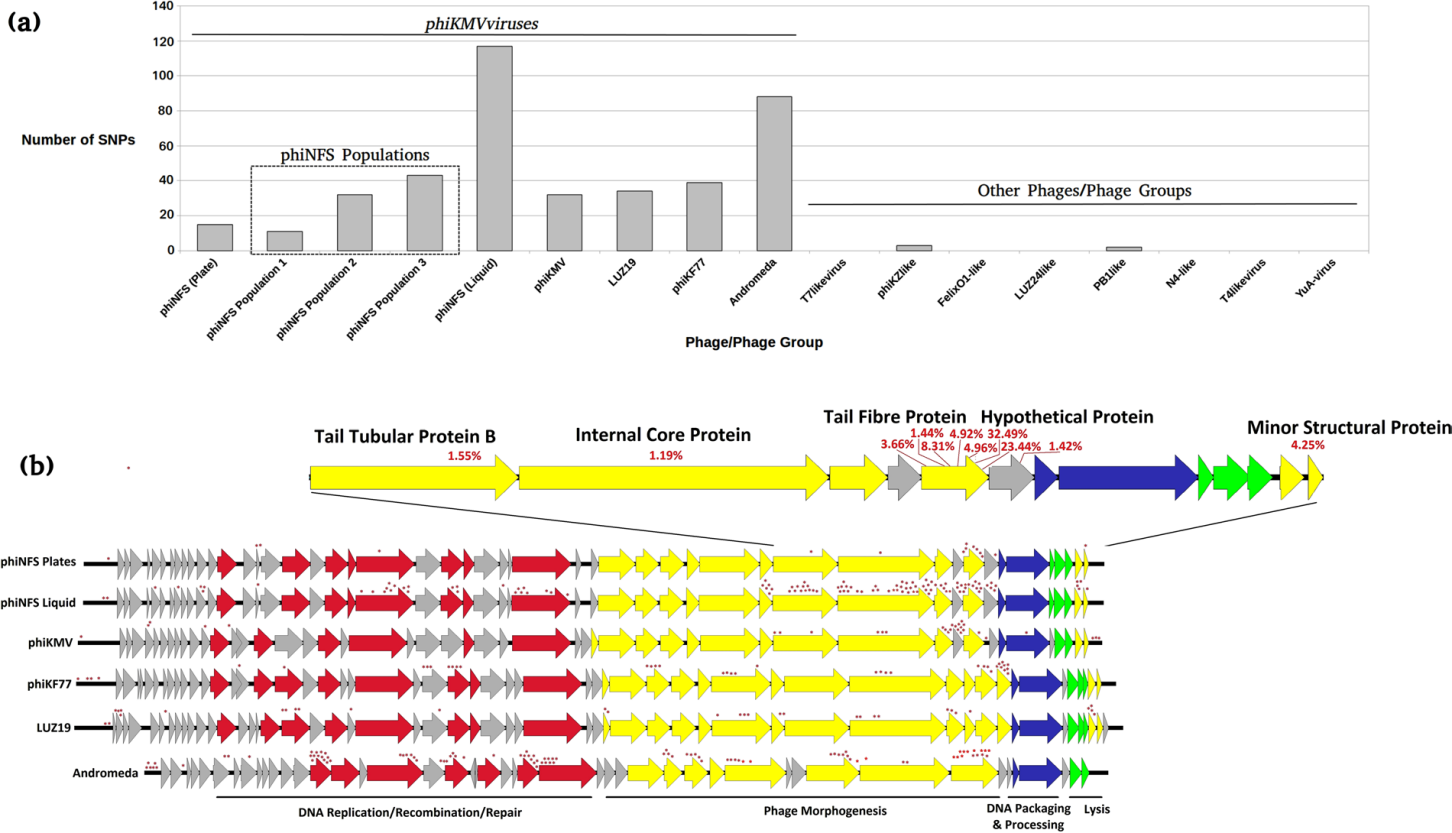
The presence and distribution of variable sites in *phiKMVviruses* and other *Pseudomonas* phage groups. (**a**) Quantitative distribution of single nucleotide variable sites amongst *phiKMVviruses* phiNFS (original sample grown on plates), three additional phiNFS (from single plaques) populations, phiNFS (grown in liquid culture), phiKMV, LUZ19, phiKF77, and Andromeda, as well as *Pseudomonas* infecting representatives from *T7virus* (DJM), *phiKZvirus* (phiKZ), *Felixounavirinae* (phiMK and phiPMW), *LUZ24virus* (phiCHU), *Pbunavirinae* (PB1), *N4virus* (phiPerm5), *T4virus* (pf16), and *YuAvirus* (phiPPE) phage groups. (**b**) Distribution of variable sites on the *phiKMVvirus* genomes analysed. Genes are coloured according to modular functions (Red: DNA replication/recombination/repair, Yellow: Structural proteins, Blue: DNA packaging and processing, Green: Lysis, and Grey: Hypothetical protein). Variable sites are represented by the red asterisks. Percentage of the substitution frequencies for the phiNFS (grown on plates) structural module variant sites are provided at their respective positions.

**Figure 3:**
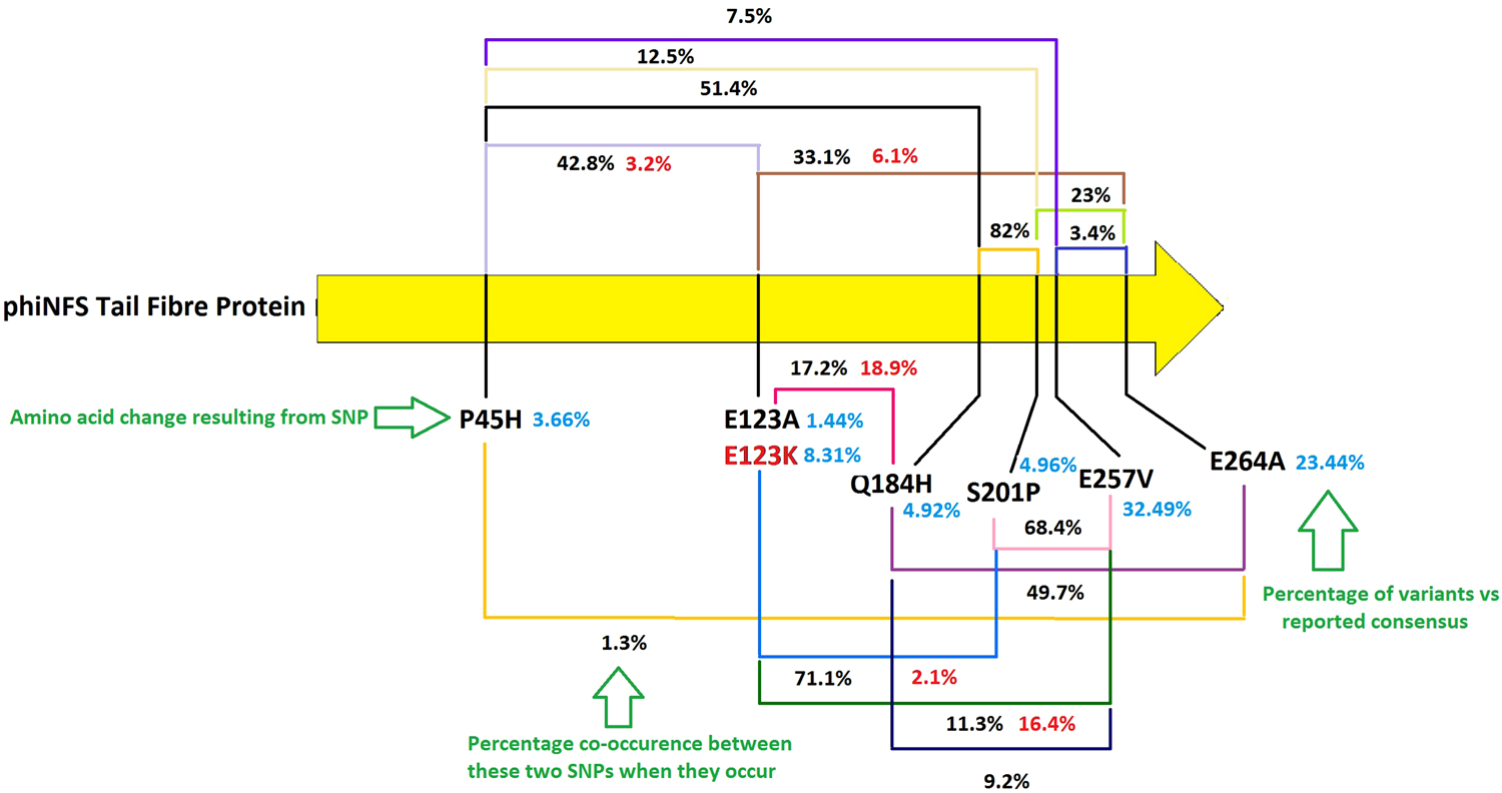
Distribution and co-occurrence of the single nucleotide variants within the phiNFS tail fibre protein gene. Percentage values in blue represent the frequency of occurrence of a single variant vs the reported consensus (KU743887). Amino acid changes associated with variants are given in black with the exception of E123K which occurs at the same position as E123A. Percentage co-occurrence values are given in black between any two linked variants with those occurring with E123K given in red.

The subsequent sequencing of phiNFS (KU743887) showed a 99% relatedness to bacteriophage phiKMV^13^, for which such behaviours have not been reported. This led to the hypothesis that elevated numbers of population variants may be responsible for the interactions observed. Analysis of Illumina data of the phiNFS genome (>6000× per base coverage), revealed the presence of 15 single base variant sites, each showing one alternative nucleotide substitution from the reported consensus sequence and existing within the phiNFS population at >1% threshold (Fig. 2a,b). The majority of these variants are AT to GC transitions. Remarkably, 11 of the 15 variants exist within the structural module of the phage genome, including 7 in the tail fibre protein (Figs 2b and 3), the major host tropism determinant^15^. One codon within the tail fibre protein contains two SNPs and displays two amino acid variants within the population, resulting in the changes E123A (1.44%) and E123K (8.31%) (Fig. 3). These are markedly diverse substitutions. In addition, whilst all variants were detected at a greater than 1% threshold, some are actually present in more than 40% of the population. In-depth analysis of the tail fibre also revealed varying levels of co-occurrence between SNPs (Fig. 3). The significance of this is that we are not observing a small number of isolated variations, but a heterogeneous assemblage of population variants reminiscent of the quasispecies phenomenon exhibited by RNA and ssDNA viruses, albeit limited to a part of the phage genome. This likely represents a compromise between adaptability and retention of the stability of having dsDNA as the hereditary molecule.

Those variants affecting non-tail fibre structural proteins possibly also have an effect on phage adsorption. It is reported that constituents other than the tail fibre protein play a role in *phiKMVvirus* adsorption^14–16^. Notably, there are a limited number of SNPs lying outside the structural module, in particular two present within gp15 at high abundance in the population (47.2% and 46.8).

Other representatives of the *phiKMVviruses* were similarly analysed: phiKMV^13^, phiKF77 (NC_012418), LUZ19 (NC_010326), and the first *phiKMVvirus* infecting *Pseudomonas syringae* known as Andromeda (NC_031014)). The *P*. *putida* KT2440 *T7virus* DJM - a non-homologous phage within the same subfamily as the *phiKMVviruses*, unrelated phages phiMK (NC_031110) and phiPMW^17^ (both *Felixounavirinae*), phiCHU^18^ (*Luz24virus*), PB1 (NC_011810)(*Pbunavirus*), phiKZ^19^ (*phiKZvirus*), pf16^17^ *(T4virus*), phiPPE (*YuAvirus*) and phiPerm5 (*N4virus*) were also analysed.

It was subsequently discovered that SNPs are prevalent throughout the phiKMV phages tested (Fig. 2a,b). The largest numbers were found within Andromeda, which possessed 88 variable sites. Again, AT to GC transitions within the structural module predominated.

Interestingly, in phage phiKMV we observe a majority of SNPs not in the same tail fibre constituent as described for phiNFS, but in the preceding hypothetical protein (gp40). It is likely that given the localisation of this protein between two genes coding for constituents of the tail fibre complex, that this also encodes a tail constituent protein. This protein (gp40), although divergent, was classified as a component of the tail fibre complex in closely related phages LUZ19 (NC_010326), MPK6 (NC_022746), and vB_Pae-TbilisiM32 (KX711710).

Upon investigating variants occurring outside of the structural module, we found a number of instances whereby conserved early proteins are subject to change (Fig. 2b). For example, in the original phiNFS sample, one variation was observed within the DNA polymerase, whereby we detected a codon change from GTG to GTA. This is in fact a silent mutation, not affecting the valine at this position. In phage Andromeda on the other hand, the wider distribution of the variants is a stark result. Looking specifically at some of these changes, there are 6 variant positions observed in the DNA polymerase. Of these, only one is a silent mutation. The other 5 variants result in changes across the amino acid range 674–681 of the polymerase. The clustering of these variants is intriguing and is a pattern that is repeated in other proteins such as the RNA polymerase. This may reflect tolerability for mutations in particular regions or that variation in these proteins is due to selective pressures for more efficient polymerase activity. The general lack of variants in a significant number of genes across all phages however, is likely due to their deleterious nature.

**Figure 4:**
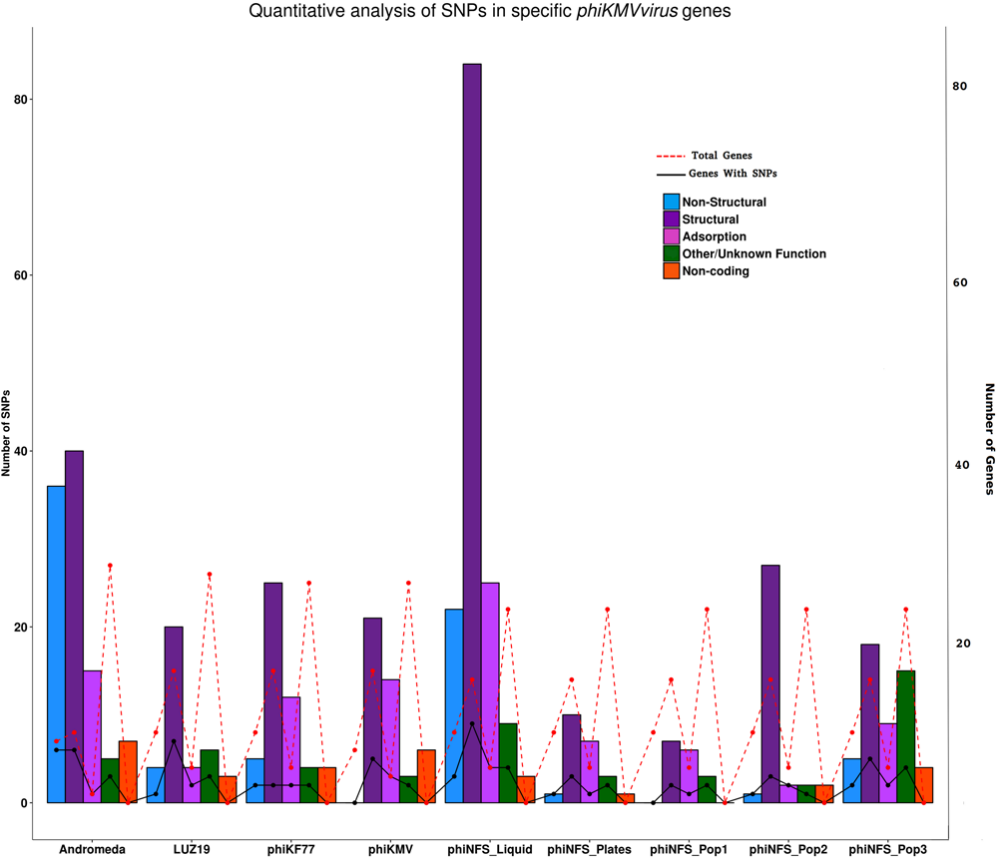
Quantitative Analysis of the Genome Wide Distribution of *phiKMVvirus* variants. Number of SNPs in each gene category (Non-structural, structural, structural associated with adsorption, Other/Unknown Function/Non-coding) are present as a clustered bar plot for each phage. Gene categories are coloured as shown in Figure. The total number of genes for each category is provided as a red dashed line. The number of genes in each category that contain at least one SNP are represented by the black line.

From a quantitative perspective, the number of variants amongst phages was investigated within the non-structural genes (DNA polymerase, RNA polymerase etc), structural genes, structural genes associated with adsorption (gp39-gp42 in phiNFS and its equivalents in other phages, gp39 only in Andromeda), genes with other functions or unknown functions, and non-coding regions, in order to gain a precise insight into any genome biases of the variants (Fig. 4). In all phages, the structural genes possess the greatest number of SNPs, with only Andromeda showing almost as many SNPs in the non-structural genes. When we observe the ratio of genes possessing SNPs compared, to the total genes for each category (Fig. 4) we observe that almost equivalent proportions of structural and non-structural genes possess variants however, almost all adsorption associated genes contain SNPs. What this analysis shows (Figs 2 and 4) is: (i) numerically, SNP abundance is greater in the structural genes of phages; (ii) with respect to proportions of genes containing at least one SNP in each category, this is almost equivalent for structural and non-structural genes; (iii) almost all adsorption associated genes contain at least one SNP.

With respect to other phages, the result is quite striking. Only PB1 and phiKZ possessed variants, with 2 and 3 respectively (1.1% and 12.33% frequencies for PB1, and 1.03%, 7.48%, and 1.03% for phiKZ). It is worth noting that both phages have much larger genomes than the *phiKMVviruses*. The lack of SNPs in DJM is also highly significant due to this phage’s close phylogenetic relationship with the *phiKMVvirus* genus.

In addition, sequencing was performed on phiNFS grown in liquid culture and on plates. The number of SNP sites in phiNFS grown in liquid medium was 3 times that from plate grown phage (Fig. 2b). This is may be due to the increased diffusion of phage and bacteria under such conditions. Therefore the “cloud” of susceptible variants of the host that is accessible by those phages capable of infecting them increases, and thus a greater proportion of phage variants are amplified and become detectable. These data suggest that selection may be driven by host physiological variations and that this may be the causative factor for the accumulation of phage variants.

In order to gain an insight into what may be happening at the population level, phiNFS originating from three individual plaques were analysed. The isolated populations possessed 11, 32, and 43 variations with a prevalence of 3.3–82.3%, the latter clearly representing a dominant variation compared to the wild-type sequence. Again, the distributions of the variants showed a similar pattern in respect to various classes of genes (Fig. 4), though there are clearly differences in prevalence, frequency, and position of these variations compared to the original phiNFS sample. What this, and the variations seen across all *phiKMVviruses* implies, is that there is no highly specific localisation of variants, rather a random generation followed by selection according to the variations in the host in each population. Given that the most amenable mechanism of resistance to a phage that a host can develop is that of receptor variability and changes in accessibility, it is not hugely surprising that the vast majority of variations observed are adsorption-associated.

**Figure 5:**
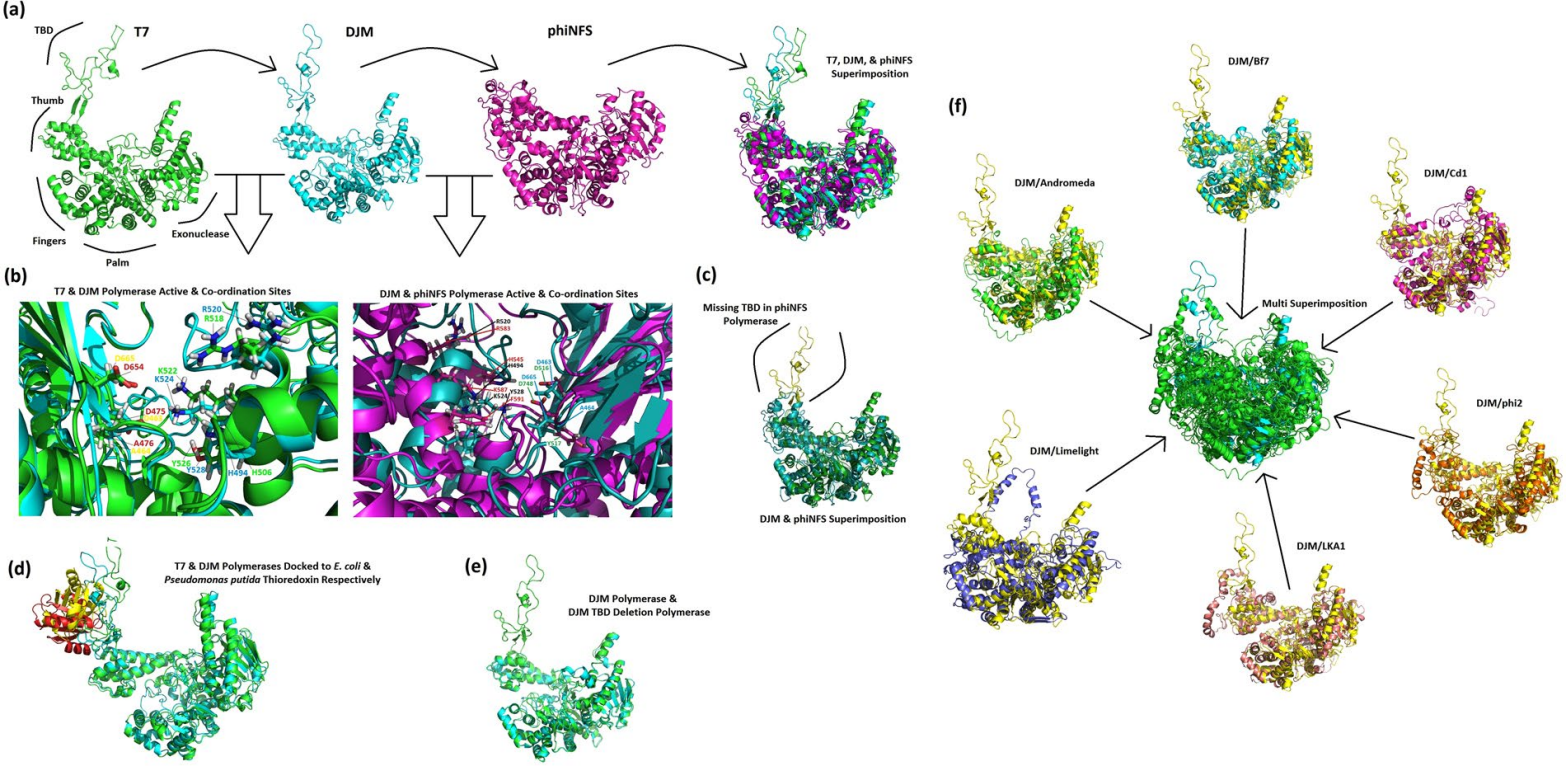
Molecular modelling and analysis of DNA polymerases from *T7viruses* T7 and DJM, and the *phiKMVvirus* phiNFS. (**a**) Molecular models of the T7 (Green), DJM (Blue), and phiNFS (Pink) DNA polymerases including with the superimposition of all three. (**b**) Conservation of the active and co-ordination site residues between phages T7 (Green) and DJM (Blue)(left) and conservation of equivalent sites between DJM (Green) and phiNFS (Blue)(right). (**c**) Superimposition of DJM (Green) and phiNFS (Blue) polymerases highlighting the absence of the thioredoxin binding domain in phiNFS (highlighted in yellow and enclosed in brackets). (**d**) Molecular docking of thioredoxin proteins from *Pseudomonas putida* (Red) and *Escherichia coli* (Yellow) to DJM (Blue) and T7 (Green) DNA polymerases respectively. (**e**) Molecular model of DJM DNA polymerase with deletion of thioredoxin binding domain (Blue), superimposed on the native DJM polymerase (Green). (**f**) Modelling of the DNA polymerases from *phiKMVviruses* Andromeda, Bf 7, Cd1, Limelight, LKA1, and phi2, all superimposed on DJM (Yellow in all instances), showing the lack of an equivalent thioredoxin binding domain across diverse members of the genus. The complete superimposition of all phages (all in green) on DJM (Blue) is given in the central portion.

With respect to a potential mechanism responsible for the generation of the variants discovered, logic dictates that the most probable route is via error prone DNA polymerase activity. The fact that an analogous strategy is utilised by RNA viruses reinforces this idea^20^. Comparisons between the crystal structure of the *Enterobacteria* phage T7 DNA polymerase (PDB code: 1skr) and molecular models of the DJM and phiNFS polymerases, revealed markedly similar structures and conservation of equivalent active sites and Mg^2+^ ion coordination sites (Fig. 5a,b). A notable difference however is observable in the phiNFS polymerase – the absence of the thioredoxin binding domain (TBD) (Fig. 5c). The absence of the TBD is also observed for closely related phages including phiKMV, which differs only in 6 of the terminal 44 amino acids of the polymerase. This domain is known to bind the host protein thioredoxin, as shown in Fig. 4d for T7 and DJM, resulting in significant increases in processivity and fidelity^21–23^. Loss of this domain has no impact on the structure of the residual DJM polymerase, highlighting that this deletion is a tolerable evolutionary change (Fig. 5e). It therefore seems probable that the absence of this domain in phiNFS, and indeed in other representatives of the *phiKMVviruses* is a contributory factor in the generation of high frequency replication errors (Fig. 5f). Indeed, a study involving the insertion of the TBD of bacteriophage T3 into *Taq* polymerase resulted in a sevenfold increase in fidelity, specifically with respect to a reduction in AT to GC transition mutations, the majority observed in the phages described here^24^. This may form the basis of a strategy to circumvent host resistant phenotypes. These findings may also explain the “adaptive” behaviour previously observed in phage phi2^25^.

In order to demonstrate *in trans* activity a cross infection experimental approach was adopted whereby *P*. *aeruginosa* PAO1 cells were co-infected with phiNFS and the unrelated SNP free phage phiMK at equal M.O.I. Sequencing and analysis of the genomic DNA extracted revealed the appearance of 67 variable sites scattered across the phiMK genome with frequencies between 1.4% and 21.2%. The fact that co-infection with phiNFS induced these SNPs reinforces the hypothesis of error-prone DNA polymerase activity being responsible for variant generation. In addition, this experiment highlights something even more significant. We have demonstrated trans-activity, whereby one group of phages can influence the evolutionary trajectory of another. Theoretically any phage exhibiting such an ability is capable of inducing variability in phages that happen to infect the same host. Therefore, this phenomenon is likely to be significant on a global scale.

Taken together, whilst the precise nature of selection at play within *phiKMVvirus* populations is not clear, it is known that co-evolution shapes many phage-host systems. Therefore, it is not improbable that the evolutionary phenomenon described here could form part of a co-evolutionary adaptation to host resistant phenotypes.

In conclusion, our results present the initial characterisation of a novel phenomenon that adds a further element of complexity to the subject of viral evolution.

## Methods

### Phage Growth and Isolation

Bacteriophages phiNFS, phiKMV, LUZ19, phiKF77, phiKZ, PB1, phiMK, phiPPE, and phiPerm5 were propagated on *Pseudomonas aeruginosa* PAO1 at 37 °C. PhiCHU was grown on *Pseudomonas aeruginosa* clinical isolate 80867 at 37 °C. Phages phiPMW and pf16 were grown on *Pseudomonas putida* PpG1 at 30 °C, and phage Andromeda and DJM grown on *Pseudomonas syringae* and *Pseudomonas putida* KT2440 respectively at the same temperature. Host populations were grown until OD = 0.3 and inoculated with their respective phage at MOI = 0.01. For liquid cultures, shaking with aeration ensued until lysis was achieved followed by overnight precipitation with polyethylene glycol 8000 at 4 °C. For phages grown on plates, 20 plates of phage at the predetermined optimal dilution were grown via the agar overlay method and the top layer removed into 4 ml of modified SM buffer (100 mM NaCl, 8 mM MgSO4, 50 mM Tris-Cl, 10 mM CaCl2). Shaking at 4 °C overnight was performed to resuspend phages followed by centrifugation of agar debris and extraction of phages in the supernatant. All phages were then purified using isopycnic CsCl centrifugation (16 hours at 60,000 rpm in a Beckman Optima TLX ultracentrifuge with rotor head TLN100) followed by extraction and dialysis.

### Co-infection of Pseudomonas aeruginosa with phiNFS and phiMK

5 ml of *Pseudomonas aeruginosa* PAO1 was grown until OD600 = 0.3. Equal titres of bacteriophages phiNFS and phiMK were pre-mixed within an Eppendorf and used to inoculate PAO1 at an MOI = 5 to permit infection with more than one phage particle. Shaking with aeration ensued until lysis was achieved and the resulting phage population extracted as supernatant following centrifugation (5 min at 10,000 × g). Plating and observation of typical phiNFS and phiMK plaques confirmed the perpetuation of both phages (or their variants). 200 ml of PAO1 (OD600 = 0.3) was inoculated with a MOI = 0.01 (from relative titre of total phages) and shaken with aeration until lysis in order to amplify the phage population. Plating again confirmed the presence of both plaque morphologies prior to purification of particles as above.

### DNA Extraction and Sequencing

DNA was extracted from phage particles using proteinase K and SDS followed by phenol-chloroform extraction as described in Sambrook and Russell^26^. DNA was purified using the MolBio DNA purification kit according to the manufacturer’s instructions. DNA was subsequently checked for purity using the NanoDrop 1000 followed by quantification using the Quantus fluorometer prior to sequencing.

Sequencing libraries were prepared from 50 ng of phage genomic DNA using the Nextera DNA Sample Preparation Kit (Illumina, USA) at the University of Cambridge Sequencing Facility. A 1% PhiX v3 library spike-in was used as a quality control for cluster generation and sequencing. Sequencing of the resulting library was carried out from both ends (2 × 300 bp) with the 600-cycle MiSeq Reagent Kit v3 on MiSeq (Illumina, USA) and the adapters trimmed from the resulting reads at the facility.

### Processing of Reads and Sequence Assembly

Initial quality checking of reads was carried out using FastQC^27^ followed by quality trimming with Trimmomatic with removal of reads with an average Q score < 20 across a 5 base sliding window^28^. Sequence assembly was subsequently carried out using SPAdes v3.6.2 and mapping of reads to the assembly carried out in order to check the fidelity of assembly^29^. Assembly of all genomes was carried out to eliminate false variants being called which may actually represent majority changes within the population compared to the reference sequence on NCBI.

### Variant Calling and Co-occurrence analysis

Reads with an average Q-Score < 30 and with length <50 were removed in order to minimise the risk of false positive results arising due to base errors and difficulties with the mapping of very short reads. Following this, an in-house variant calling pipeline was utilised as follows: mapping of reads to reference genomes was conducted separately with Bowtie2, BWA, and BBMap^30–32^. This is due to the differences in the output of various mapping tools. Each output was taken through Samtools indexing and conversion to produce a final BAM file upon which VarScan and FreeBayes were utilised separately to call variants^33,34^. Variants were called only on positions of the genome with a minimum of ×100 per base coverage. The inclusion criterion for all six values was set as being within two standard deviations of the mean. No values fell outside of this, and were all in fact within a minimum range of 1% of each other. Following the variant calling pipeline, BAM files were loaded into IGV and manual inspection of the variant sites carried out at the reported genomic loci in order to confirm their presence.

Co-occurrence analysis of SNPs was carried out with an in-house Python script which provides the percentage ratio of reads containing one or both variants of two polymorphisms of interest.

### Molecular Modelling

Molecular modelling of DNA polymerases was carried out using the I-Tasser server followed by energy minimisation using the Yasara energy minimisation server of the most favourable prediction^35,36^. Stereochemical clashes and model fidelity were checked using Chimera and the Whatif server tools^37^. In addition, Ramachandran analysis was carried out as an additional assessment of model fidelity. Models were viewed in PyMol and alignments carried out using the TM-align algorithm^38,39^.
